# Supplementary material for: ERα promotes murine hematopoietic regeneration through the Ire1α-mediated unfolded protein response
Source: eLife. 2018 Feb 16;7:e31159. doi: 10.7554/eLife.31159 (PMC5829925; doi:10.7554/eLife.31159)
Supplement: Supplementary file 1. — All primers are listed in the 5’ to 3’ direction. [file elife-31159-supp1.docx]

**Table S1. List of primers used for qPCR and CRISPR editing**

| Target | Sequence |
| --- | --- |
| B-actin F | CGTCGACAACGGCTCCGGCATG |
| B-actin R | GGGCCTCGTCACCCACATAGGAG |
| Ern1 F | CTTGAGGAATTACTGGCTTCTCA |
| Ern1 R | TCCAGCATCTTGGTGGATG |
| CHOP/Ddit3 F | CATGTTGAAGATGAGCGGGTG |
| CHOP/Ddit3 R | TGGAACACTCTCTCCTCAGGT |
| ATF6 - F | GGACGAGGTGGTGTCAGAG |
| ATF6 - R | GACAGCTCTTCGCTTTGGAC |
| PERK - F | CCTTGGTTTCATCTAGCCTCA |
| PERK - R | ATCCAGGGAGGGGATGAT |
| Xbp1 Taqman F | AGTGGAGTAAGGCTGGTGGC |
| Xbp1 Taqman R | TGAAGAGGCAACAGTGTCAGAG |
| Hsp90aa1 F | GTGTTCATTCAGCCACGATG |
| Hsp90aa1 R | CTGCCTGAAAGGCAAAGGT |
| Dnajc18 F | CATTGATTACATCCAGACGAGTTG |
| Dnajc18 R | AATTGGTCAGCTCCGACTTTT |
| Hspa1b F | AACCAGTATGTTCTTTGCATTTAATC |
| Hspa1b R | TGTACACAGGGTGGCAGTGT |
| Slc7a5 F | ATGTGGCTCCGATTCAAGA |
| Slc7a5 R | GGAGGGCCAGATTCACCT |
| Ifit1 F | TCTAAACAGGGCCTTGCAG |
| Ifit1 R | GCAGAGCCCTTTTTGATAATGT |
| Pdia5 F | AAGTGCGAGGCCACATTG |
| Pdia5 R | TGTTCTCGAACTCTGATGGGTA |
| Lsm1 F | TTCCTCGAGGGATTTTCGT |
| Lsm1 R | GTCACTCTCCTTCTCCAGGTCTA |
| Exosc9 F | CCCCTATTGATACCTCCAACAT |
| Exosc9 R | CCACAGCACAGGTTGAGAAA |
| Vegfa F | AAAAACGAAAGCGCAAGAAA |
| Vegfa R | TTTCTCCGCTCTGAACAAGG |
| Sec11a F | CCATCTCACCATGCTGTCTC |
| Sec11a R | CGAGGAGACAATCATTCCAAA |
| Spcs1 F | TGAACAGTTTGGGTGGACTGT |
| Spcs1 R | CCATGGAGGAAGTGTCAGC |
| Hspa9 F | TGCAGAGAAGTACGCTGAGG |
| Hspa9 R | GCCATATTAACTGCTTCAACACG |
| Dnaja1 F | TGGCTCTGCAAAAGAATGTG |
| Dnaja1 R | TGAATCCTTATCTGCATACCTGTC |
| Hspa8 F | GCAGCTGGGCCTACACAC |
| Hspa8 R | GGCAATAATTTCCACCTTTCC |
| Rosa26 sgRNA | taatacgactcactataGGAGCCACCCTACAAGAGGTgtttaagagctatgctggaaacagc |
| IRE1α sgRNA1 | taatacgactcactataGGAAATTTCATTCTGCCCCAgtttaagagctatgctggaaacagc |
| IRE1α sgRNA2 | taatacgactcactataGGCCCTCAGCTCCATGACCCgtttaagagctatgctggaaacagc |
| IRE1α sgRNA3 | taatacgactcactataGGAGCCACCCTACAAGAGGTgtttaagagctatgctggaaacagc |
| sgRNA reverse | AGCACCGACTCGGTGCCACT |
| Tipin F | TGCCTATTGTACATGAAGATTTTGT |
| Tipin R | TGTTAGGCTTCTAGTTGGTTCAGA |
| Tyms F | TGCTCACCTACATGATTGCAC |
| Tyms R | TCTCCCAAAGTGTGGACAAA |
| Ccna2 F | GTCAACCCCGAAAAACTGG |
| Ccna2 R | AAGGTCCTTAAGAGGAGCAACC |
| Pole3 F | TCCAGCGATTCATAACACCA |
| Pole3 R | GCTCCGAAGCCTCCTTCT |
| Hmga1 F | AGACCCGGAAAGTCACCA |
| Hmga1 R | CCTCCTCTTCCTCCTTCTCC |
| Aimp2 F | GACTTGAATTCCGTGCTTGG |
| Aimp2 R | CACAAGCAGGGACAGTGGT |
| Cdkn2c F | AAATGGATTTGGGAGAACTGC |
| Cdkn2c R | CAAATTGGGATTAGCACCTCTG |
| Ire1α-sg2 upstream F | GAGCCCTGACATTCTTAAAG |
| Ire1α-sg3 downstream R | CGTGAAGAGGATCCTCCCTG |
| Ire1α ChIP P1 F | GTTGTTTTGTGGGCGTGGTAA |
| Ire1α ChIP P1 R | GCAGCTAGAAGCTAGCACCG |
| Ire1α ChIP P2 F | AGAGGGGAAATTGCTGGGCT |
| Ire1α ChIP P2 R | TCTGCTACCTTTACAGGGTCACT |
| Ire1α ChIP P3 F | GTTTTACACTTCCCACAGGTGC |
| Ire1α ChIP P3 R | CTCAATGCCAGGCCGTTTG |
