# Supplementary material for: ERα promotes murine hematopoietic regeneration through the Ire1α-mediated unfolded protein response
Source: eLife. 2018 Feb 16;7:e31159. doi: 10.7554/eLife.31159 (PMC5829925; doi:10.7554/eLife.31159)
Supplement: Transparent reporting form [file elife-31159-transrepform.pdf]

1st Floor
24 Hills Road
Cambridge CB2 1JP, UK

P 01223 855340
W elifesciences.org
T @elife

eLife's transparent reporting form

We encourage authors to provide detailed information *within their submission* to facilitate the interpretation and replication of experiments. Authors can upload supporting documentation to indicate the use of appropriate reporting guidelines for health-related research (see EQUATOR Network), life science research (see the BioSharing Information Resource), or the ARRIVE guidelines for reporting work involving animal research. Where applicable, authors should refer to any relevant reporting standards documents in this form.

If you have any questions, please consult our Journal Policies and/or contact us: editorial@elifesciences.org.

Sample-size estimation

- You should state whether an appropriate sample size was computed when the study was being designed
- You should state the statistical method of sample size computation and any required assumptions
- If no explicit power analysis was used, you should describe how you decided what sample (replicate) size (number) to use

Please outline where this information can be found within the submission (e.g., sections or figure legends), or explain why this information doesn't apply to your submission:

For experiments that involved collecting sample materials from multiple mice (Fig 1a,b, 4e,f,g, 5f, S4a,b,c,d,f, S5a,b), three independent replicates were obtained to allow statistical inference while using a justifiable number of animals. For other experiments, we aimed for 4-6 biological replicates per group. For most experiments, given a standard deviation of approximately 30% (based on previous experiments), this sample size allowed us to determine changes of 55% in the mean with a power of 80% in two-tailed t tests (alpha = 0.05). In some instances, larger sample sizes were obtained by collecting observations from mice in previous experiments (Fig 1e-j).

Replicates

- You should report how often each experiment was performed
- You should include a definition of biological versus technical replication
- The data obtained should be provided and sufficient information should be provided to indicate the number of independent biological and/or technical replicates
- If you encountered any outliers, you should describe how these were handled
- Criteria for exclusion/inclusion of data should be clearly stated
- High-throughput sequence data should be uploaded before submission, with a private link for reviewers provided (these are available from both GEO and ArrayExpress)

Please outline where this information can be found within the submission (e.g., sections or figure legends), or explain why this information doesn't apply to your submission:

eLife Sciences Publications, Ltd is a limited liability non-profit non-stock corporation incorporated in the State of Delaware, USA, with company number 5030732, and is registered in the UK with company number FC030576 and branch number BR015634 at the address 1st Floor, 24 Hills Road, Cambridge CB2 1JP | August 2014

Samples sizes are described in the Figure Legends.

Specifically, sample sizes for each data set are as follows. Fig. 1a: n = 3 biological replicates. Fig. 1b: n = 6 (3 biological replicates, 2 technical replicates per mouse). Fig. 1c: n = 10 (2 biological replicates, 5 fields of view per bone). Fig. 1e-j: n = 11 (Oil ERα-KO, 6 recipients per donor), n = 18 (E2-ERαKO, 6 recipients per donor), n = 24 (Oil and E2 WT, 6 recipients per donor). Fig. 2a-c: n = 4 biological replicates. Fig. 2d-f: n = 4 (Oil WT, Oil ERα-KO, E2 ERα-KO), n = 5 (E2 WT). Fig. 2g: n = 7 (E2), n = 9 (Oil). Fig. 2h: n = 9 (Female), n = 10 (Male). Fig. 3a-c: n = 4 (Oil and E2 Day 3,5, and 14), n = 6 (Oil and E2 Day 7). Fig. 3d: n = 5 (Unirradiated, 1 donor), n = 7 (Oil, 4 recipients per donor), n = 8 (E2, 4 recipients per donor). Fig. 3e-g: n = 3 (E2 Unirradiated), n = 4 (Oil ERα-KO TBI), n = 5 (E2 ERα-KO TBI), n = 6 (Oil Unirradiated, E2 TBI), n = 7 (Oil TBI). Fig. 4a: n = 8 (2 biological replicates per group). Fig. 4e: n = 3 biological replicates. Fig. 4f: n = 3 biological replicates. Fig. 4g: n = 3 biological replicates. Fig. 5a: n = 5 biological replicates. Fig. 5b: n = 4 (E2), n = 5 (Oil). Fig. 5d: n = 3 (E2 Unirradiated, E2 TBI), n = 4 (Oil TBI), n = 5 (Oil Unirradiated). Fig. 5f: n = 3 biological replicates. Fig. 5g: n = 7 (sgRNA-Ire1 E2), n = 9 (sgRNA-Rosa26 Oil and E2), n = 10 (sgRNA-Ire1 Oil). Fig. S1c: n = 8 (E2, 4 recipients per donor), n = 11 (Oil, 4 recipients per donor). Fig. S2a-d: n = 4 (Oil Unirradiated), n = 5 (Oil and E2 TBI). Fig. S2e-f: n = 4 (Oil and E2 Day 3,5, and 14), n = 6 (Oil and E2 Day 7). Fig. S2g: n = 3 (E2 Unirradiated), n = 4 (Oil ERα-KO TBI), n = 5 (E2 ERα-KO TBI), n = 6 (Oil Unirradiated, E2 TBI), n = 7 (Oil TBI). Fig. S2h: n = 3 (Unirradiated control), n = 4 (Oil and E2 TBI Days 3 and 5). Fig. S3a: n = 3 biological replicates. Fig. S4a: n = 3 biological replicates. Fig. S4b: n = 3 biological replicates. Fig. S4c: n = 3 biological replicates. Fig. S4d: n = 3 biological replicates. Fig. S4e: n = 4 biological replicates. Fig. S4f: n = 3 (G-CSF), n = 4 (Poly(I:C)). Fig. S5: n = 3 biological replicates.

Statistical reporting

- Statistical analysis methods should be described and justified
- Raw data should be presented in figures whenever informative to do so (typically when N per group is less than 10)
- For each experiment, you should identify the statistical tests used, exact values of N, definitions of center, methods of multiple test correction, and dispersion and precision measures (e.g., mean, median, SD, SEM, confidence intervals; and, for the major substantive results, a measure of effect size (e.g., Pearson's r, Cohen's d)
- Report exact p-values wherever possible alongside the summary statistics and 95% confidence intervals. These should be reported for all key questions and not only when the p-value is less than 0.05.

Please outline where this information can be found within the submission (e.g., sections or figure legends), or explain why this information doesn't apply to your submission:

Statistical analysis methods are described in each figure legends, as well as in the "Quantification and Statistical Analysis" section of the Materials and Methods. Specifically, unless indicated otherwise in the figure legends, analyses were always carried out in comparison to the control group (Oil, DMSO, etc). When comparing means from groups with unequal sample sizes, Mann Whitney tests were used for the comparison of two groups (Fig S5d), and Kruskal Wallis tests with Dunn's multiple comparison tests were used for comparing three or more groups (Fig 6e). Otherwise, two-tailed t-tests were used for the comparison of two groups (Fig. 1c, 2a-c, 3a-d, 5a, and Supplementary Fig S2f-g, i, S3a, S4b,f) and one- and two-way ANOVAs for comparisons involving more than two groups or more than two independent variables (Figs 1a-b, e-j, 2d-f, 3d-g, 4d-g, 5d-e, 6b,d and Supplementary Fig S1d, S2b-e, h, S3b-i, S4c-g, S5c-d) with Bonferroni corrections performed for multiple testing. Analysis of survival curves was performed using a log-rank test (Fig 2g-h).

(For large datasets, or papers with a very large number of statistical tests, you may upload a single table file with tests, Ns, etc., with reference to sections in the manuscript.)

Group allocation

- Indicate how samples were allocated into experimental groups (in the case of clinical studies, please specify allocation to treatment method); if randomization was used, please also state if restricted randomization was applied
- Indicate if masking was used during group allocation, data collection and/or data analysis

Please outline where this information can be found within the submission (e.g., sections or figure legends), or explain why this information doesn't apply to your submission:

This is described in the "Quantification and Statistical Analysis" section of Materials and Methods. All mice were age and sex matched for each experiment. Unless indicated otherwise only male mice were used, and littermate controls were used for ERaKO studies. Distribution of mice was not formally randomized, and no experiments were blinded.

eLife Sciences Publications, Ltd is a limited liability non-profit non-stock corporation incorporated in the State of Delaware, USA, with company number 5030732, and is registered in the UK with company number FC030576 and branch number BR015634 at the address 1st Floor, 24 Hills Road, Cambridge CB2 1JP | August 2014

Additional data files ("source data")

- We encourage you to upload relevant additional data files, such as numerical data that are represented as a graph in a figure, or as a summary table
- Where provided, these should be in the most useful format, and they can be uploaded as "Source data" files linked to a main figure or table
- Include model definition files including the full list of parameters used
- Include code used for data analysis (e.g., R, MatLab)
- Avoid stating that data files are "available upon request"

Please indicate the figures or tables for which source data files have been provided:

No source data files are provided.
